# Comparison of the Efficacy and Safety of Insulin Glargine and Insulin Detemir with NPH Insulin in Children and Adolescents with Type 1 Diabetes Mellitus Receiving Intensive Insulin Therapy

**DOI:** 10.4274/jcrpe.v1i4.56

**Published:** 2010-12-08

**Authors:** Bumin Nuri Dündar, Nihal Dündar, Erdal Eren

**Affiliations:** 1 Department of Pediatric Endocrinology, Süleyman Demirel University, Faculty of Medicine, Isparta; 2 Department of Pediatrics, Süleyman Demirel University, Faculty of Medicine, Isparta; 3 Department of Pediatric Endocrinology, Uludağ University, Faculty of Medicine, Bursa; +90-246-211 22 76+90-246-211 22 09bumindundar@gmail.comSuleyman Demirel University, Faculty of Medicine Department of Pediatric Endocrinology Cunur-32260-Isparta, Turkey

**Keywords:** children, adolescents, NPH, glargine, detemir

## Abstract

**Objective**: The purpose of this study was to compare the efficacy and safety of insulin glargine and detemir with NPH insulin in children and adolescents with type 1 diabetes mellitus (DM).

**Methods**: Thirty four children and adolescents with type 1 DM (mean age 12.7 ± 3.4 years, diabetes duration 5.4 ± 3.0 years) were included in the study. All patients had been receiving intensive insulin therapy with insulin aspart and NPH for at least 6 months before switching from NPH to insulin glargine (Group 1, n=19) or detemir (Group 2, n=15). The medical records obtained within 6 months before and after treatment with insulin glargine and detemir were retrospectively reviewed and the data were compared in each group.

**Results**: The mean age and duration of DM were similar in two groups (p>0.05). In both groups, switching from NPH to insulin glargine or detemir, resulted in a reduction in HbA_1c_ (p0.05, for both). Patients in the detemir treated group had less increment in body mass index (BMI) SDS at the end of 6 months of therapy compared to NPH and glargine treated patients (p>0.05, for both). No side effects were noted throughout the study.

**Conclusion**: Both insulin glargine and detemir improved HbA_1c_ at short-term and proved to be safe and well tolerated in children and adolescents with type 1 DM.

**Conflict of interest:**None declared.

## INTRODUCTION

Diabetes Control and Complications Trial (DCCT) has shown that intensive diabetes management in adults and adolescents results in better glycemic control and delays the onset of vascular and neurological complications. ^1^ Intensive diabetes mellitus (DM) management consists of multiple daily injections of both rapid and longer-acting insulin preparations which aim to mimic endogenous insulin secretion pattern characterized by continuous basal insulin secretion and meal-related peaks. In the past, human regular, NPH, and ultralente insulins have all been used as part of this regimen.^2^ Recently however, recombinant DNA technology has been used to design new insulin molecules that overcome the limitations of regular and NPH insulin. Combinations of both rapid-acting insulin analogues like insulin aspart and lispro and long acting insulin analogues (LAIA) such as insulin glargine and detemir have been incorporated into treatment regimens.^3, 4^

Insulin glargine is a relatively new, longacting insulin analogue with a COOH-terminal elongation of the B-chain by two arginines and a replacement in the A-chain of asparagine by glycine in position A21 (21A-Gly-30Ba-L-Arg-30Bb-L-Arg-human insulin). These modifications result in a shift of the isoelectric point (from a pH of 5.4 to 6.7), making the new molecule less soluble at physiological pH compared to the native insulin molecule; consequently, insulin glargine has a prolonged duration of action that lasts approximately 24 hours, with no pronounced peak in activity.^5^ Another LAIA is insulin detemir which is an acylated derivative of human insulin [Lys B29 (N^ε^ -tetradecanoyl) des (B30) human insulin]. It has more reproducible absorption and a prolonged action profile.^6^

In recent years, due to their benefits compared to NPH as basal insulin in the treatment of type 1 DM, these LAIA have been widely used in pediatric cases. However, there are few studies available in literature comparing the safety and efficacy of these insulins in children and adolescents with type 1 DM.

The aim of this study was to compare the efficacy and safety of insulin glargine and insulin detemir with NPH and with each other in children with type 1 DM who have been receiving intensive insulin therapy.

## METHODS

A total of 34 children and adolescents (19 female and 15 male) diagnosed with Type 1 DM who had regular controls at least for 1 year at our Pediatric Endocrinology Department were included in the study. The mean age of the patients and the mean duration of disease were 12.7 ± 3.4 and 5.4 ± 3.0 years, respectively. Previously, all patients had been using intensive insulin therapy with 3 doses of insulin aspart and one dose of NPH for at least 6 months. At onset of study, NPH treatment was switched to insulin glargine (Group 1, n=19) or detemir (Group 2, n=15) once daily without any change in the rapid acting insulin therapy. Insulin glargine and detemir treatments were started with a dose that was 40-45% of total daily insulin doses. Insulin to carbohydrate ratio for meals was not used during the study period. Characteristics of the patients in Group 1 and Group 2 are shown in Table 1. Four patients in group 1 (21%) and 3 patients in group 2 (20%) were prepubertal. At the onset of treatment with the new insulins, patients were informed about the principles of action for insulin glargine and insulin detemir. All patients had received the same education regarding diabetes at the onset of their disease.

The characteristics of Group 1 and Group 2 are shown in Table 1. The mean age and duration of DM were not significantly different between the two groups (p>0.05). All patients were evaluated every three months. At each visit, a comprehensive physical examination was done and patient data including insulin doses, fasting glucose levels, HbA_1c_ values, frequency of severe hypoglycemic attacks, weight and height measurements were recorded. Data pertaining to the 6 month periods before and after treatment with insulin glargine and detemir were evaluated in the two groups. 

HbA_1c_ values were measured at baseline and every 3 months, using the HPLC method (normal value 5.1 ± 0.31%). 

Severe hypoglycemia was defined as an event with symptoms consistent with hypoglycemia in which the patient required assistance from another person. The number of attacks of severe hypoglycemia and nocturnal hypoglycemia during 6 months of treatment with NPH and LAIA were recorded. Fasting glucose values were recorded from patient’s glucometer. All patients were requested to check blood glucose at least 4 times per day and the mean value was calculated. Weight and height measurements of the patients were obtained using standard devices. Body mass index (BMI) was determined using the formula: BMI= weight (kg) / height^2^ (m^2^) and BMI SDS was calculated according to normal data obtained from sex and age matched subjects.^7^ Patients were asked and examined for side effects and all adverse events were noted at clinic visits. This study has been conducted in accordance with the guidelines in the Declaration of Helsinki and has been formally approved by local institutional ethical committee.

**Statistical Analysis**

SPSS 15 software package was used for statistical analysis. Data were expressed as mean ± standard error of the mean (SEM). Statistical comparisons were performed using non-parametric Mann-Whitney U, Wilcoxon and Chi-square tests. Statistical significance was defined as p<0.05.

**Table 1:**
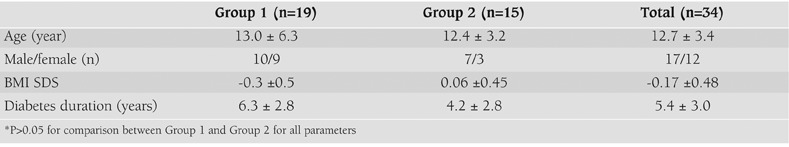
Characteristics of the patients in Group 1 and Group 2 at onset of study*

## RESULTS

The mean daily insulin requirements, the mean fasting glucose levels and the frequency of severe hypoglycemic attacks observed in Group 1 and Group 2 during 6 months of therapy with NPH and LAIA are shown in Table 2 and 3. Daily bolus and total insulin doses during NPH treatment were similar to the doses used during insulin glargine and detemir treatments (p>0.05, for both). There was also no difference in insulin doses between patients treated with insulin glargine and detemir during the study period (p>0.05). The mean fasting blood glucose levels did not change significantly during NPH and LAIA treatment periods (p>0.05, for both). The mean number of severe hypoglycemic attacks during 6 months of treatment with NPH and LAIA were not significantly different (p>0.05). The number of nocturnal hypoglycemic events were found to be reduced after treatment with LAIA therapy, but the difference was not significant (p>0.05). (Before treatment with LAIA: 5 (26%) episodes in two patients in Group 1 and 3 (15%) episodes in two patients in Group 2; after treatment with LAIA: 2 (13%) episodes in 2 patients in Group 1 and 1 (6%) episode in one patient in Group 2).

**Figure 1:**
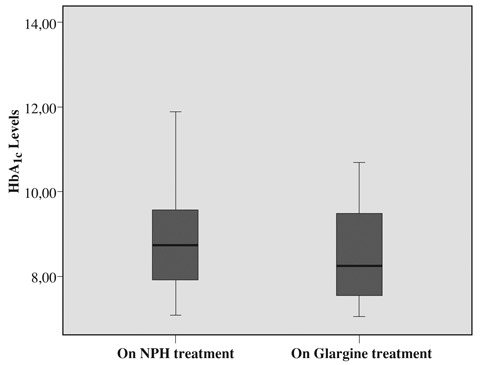
Mean HbA_1c_ values on NPH and on glargine treatment (9.21 ± 1.71 and 8.47 ± 1.04, p<0.05)

In both groups, the mean HbA_1c_ values decreased significantly from baseline after initiating LAIA therapy (p<0.05, for both) (Figure 1 and 2). However, no significant difference was found between glargine and detemir treated patients (p>0.05).

**Figure 3:**
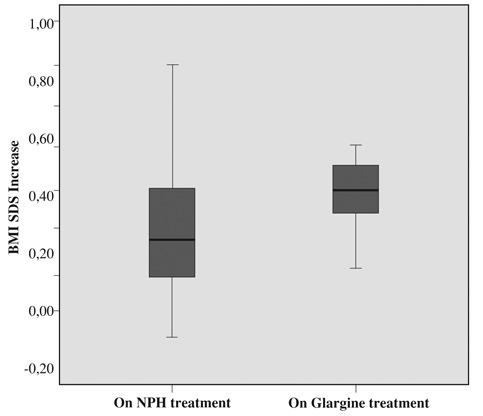
Increase in BMI SDS on NPH and on glargine treatment (0.38 ± 0.25 and 0.40± 0.25, p>0.05)

The increase in BMI SDS in detemir-treated group during 6 months was significantly lower than the increase noted in NPH and glargine-treated groups (p<0.05) (Figure 3 and 4). Pre- and post-treatment increase in BMI SDS in the glargine group was similar (p>0.05). No side effects were observed during treatment with glargine and detemir insulin and both insulins were well tolerated.

**2:**
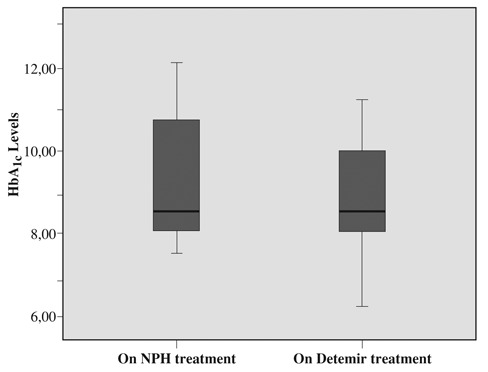
Mean HbA_1c_ values on NPH and on detemir treatment (9.26 ± 1.31 and 8.97 ± 1.4, p<0.05)

**4:**
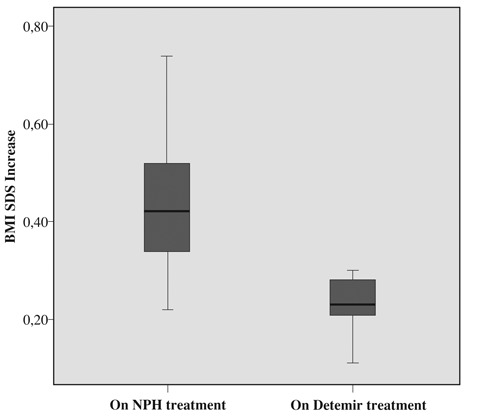
Increase in BMI SDS on NPH and on detemir treatment (0.43 ± 0.14 and 0.27 ± 0.15, p<0.05)

**Table 2:**
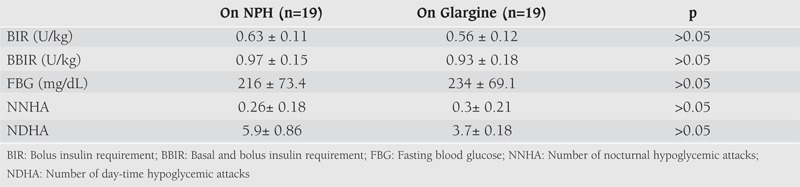
Daily insulin requirements, mean fasting blood glucose levels and number of severe hypoglycemic attacks in Group 1 on NPH and on insulin glargine

**3:**
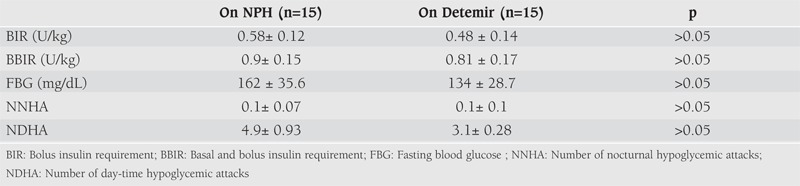
Daily insulin requirements, mean fasting blood glucose levels and number of severe hypoglycemic attacks in Group 2 on NPH and on insulin detemir

## DISCUSSION

In this study, HbA_1c_ values were found to be significantly reduced in children with type 1 DM using intensive insulin therapy after their basal insulin was switched from NPH to insulin glargine or detemir, without any significant changes in daily insulin requirements and fasting glucose levels. This result seems to be consistent with most of the previous studies investigating the effect of insulin glargine and detemir in adults and children with type 1 DM in which improvements in HbA_1c_ values were shown with or without any change in daily insulin requirements and fasting glucose levels.^8, 9, 10, 11^ On the other hand, some studies did not report any significant difference in HbA_1c_ levels between NPH and LAIA treatments; this is probably due to discrepancies in sample size, treatment regimens and patient characteristics between these studies.^12, 13^ Although the sample size of our study is small, our results indicate that both insulin glargine and detemir, administered as part of intensive insulin therapy, have a more predictable glucose- lowering effect than NPH treatment. We think that this effect is probably associated with longer duration of action of these insulins which enables them to mimic physiological patterns more closely. 

The frequency of attacks of severe hypoglycemia and the ratio of nocturnal hypoglycemic events were higher during NPH treatment in the two groups compared with respective values on LAIA therapy, but these differences were not significant. However, it is well known that LAIA shows a flat profile of plasma insulin levels and no pronounced peak of activity, both of which are characteristics associated with a lower relative risk of hypoglycemia, especially at night.^10, 11, 12, 13, 14, 15^

It has been demonstrated that glargine and detemir have different pharmacokinetic and pharmacodynamic properties.^14, 15, 16^ However; there are few studies in children and adolescents with type 1 DM comparing the effects of these insulins. Kabadi^17^ et al showed worsening in HBA_1c_ levels in patients switching from insulin glargine to insulin detemir twice a day. In a study on adult patients, Meneghini at al^18^ reported that HbA_1c_ was significantly reduced from baseline after starting insulin detemir in patients with type 2 DM who have previously received NPH and glargine insulin. Monami et al^19^ on the other hand showed no significant differences in a similar group of patients. Although insulin requirements, fasting glucose levels and HbA_1c_ values during treatment were similar in glargine and detemir treated groups during the study period in the present study, there is a need for randomized, double-blind and controlled studies to compare the effects of these insulins in children with type 1 DM.

The mean BMI SDS increase in the detemir treated group was found to be lower than the increase in patients treated with NPH and glargine in this study. In previous studies, a weight advantage which could not be explained by BMI-related differences has also been reported with insulin detemir compared to NPH insulin.^13, 20, 21^ A number of hypotheses have been suggested to explain this observation.^22, 23^ The first, concerns the risk reduction for hypoglycemia typically observed with insulin detemir and it has been suggested that reduced nocturnal hypoglycemia may reduce the need for additional precautionary calorie intake by the patient.^24^ Another potential explanation for the weight-sparing property of insulin detemir is that it might exert a greater effect on endogenous hepatic glucose production relative to peripheral glucose uptake, compared to subcutaneously injected human insulin.^25^ This would imply that insulin detemir could exert a relatively lower inhibitiory effect on peripheral lipolysis for a given total glucose-lowering effect.

Injection site reactions have been reported, particularly during insulin detemir administration. However, no side effects were observed in our patients. Both insulins were well tolerated during the study period. Nevertheless, long-term adverse effects of insulin glargine and detemir in children are still unknown.

In conclusion, our results indicate that using insulin glargine or detemir instead of NPH insulin in the treatment of children and adolescents with type 1 DM receiving intensive insulin management, improves metabolic control with similar efficacy and without increasing the number of hypoglycemic events. Insulin detemir seems to have the additional advantage of producing less weight gain. Further carefully designed, long-term, prospective studies are needed to evaluate the overall benefits and clinical efficacy of LAIA therapy in children and adolescents with type 1 DM.
